# Prognostic Significance of Histopathological Parameters for Salivary Gland Adenoid Cystic Carcinoma

**DOI:** 10.3390/dj11110262

**Published:** 2023-11-09

**Authors:** Everton Freitas de Morais, Hannah Gil de Farias Morais, Roseana de Almeida Freitas, Ricardo D. Coletta

**Affiliations:** 1 Department of Oral Diagnosis, School of Dentistry, University of Campinas, Piracicaba 13414-018, SP, Brazil; evertonf@unicamp.br; 2 Postgraduate Program in Oral Science, Federal University of Rio Grande do Norte, Natal 59000-000, RN, Brazil; hannah.gil.102@ufrn.edu.br (H.G.d.F.M.); roseana.freitas@ufrn.br (R.d.A.F.); 3 Department of Oral Diagnosis and Graduate Program in Oral Biology, School of Dentistry, University of Campinas, Piracicaba 13414-018, SP, Brazil

**Keywords:** malignant salivary gland tumors, prognosis, histopathological features, histopathological grading systems

## Abstract

Adenoid cystic carcinoma (ACC) is a rare salivary gland tumor that accounts for approximately 1% of all head and neck cancers. Despite its initial indolent behavior, long-term survival is poor due to locoregional recurrence in approximately 40% and distant metastasis in up to 60% of patients who undergo radical treatment. The histological parameters of ACC and the combination of these parameters in histopathological grading systems provide valuable prognostic information about the clinical course of the disease. Within this context, this review aims to analyze the impact of histopathological parameters, individual or combined in histopathological grading systems of malignancy, on ACC prognosis. Individual histopathological parameters such as solid pattern, presence of tumor necrosis, high-grade transformation, dominance of the epithelial component, presence of perineural and lymphovascular invasion, and positive surgical margins have negative impacts on the survival of patients with ACC. There are currently four histopathological grading systems for ACC; however, few studies have validated these systems and most of them explored small cohorts with short follow-up. Considering that the application of grading systems has been associated with ACC prognosis, a broader validation will allow not only their use for prognostic prediction but also assist in treatment planning.

## 1. Introduction

Malignant salivary gland tumors, whose global annual incidence rate ranges from 0.05 to 4.5 cases per 100,000 persons, comprise a heterogeneous group of tumors that differ not only in terms of histopathological characteristics but also in their biological behavior [1]. This heterogeneity determines a classification based on 22 different malignant neoplasms [2]. Adenoid cystic carcinoma (ACC) is a glandular malignant tumor that can affect different anatomical sites, including the major and minor salivary glands, lacrimal glands, and glands of the upper digestive tract [3]. In the head and neck region, ACC accounts for 1% of all cancers and for 10% of salivary gland tumors [4]. Despite its slow growth, the prognosis of ACC is poor due to its local invasiveness and high rates of recurrence [4,5]. In a series of 105 patients, van Weert et al. [6] reported survival rates after 5, 10, and 20 years of 68%, 52%, and 28%, respectively. The poor long-term survival rate of ACC is associated with the difficulty in controlling distant metastases, which occur more frequently in the lungs [7,8,9].

The differential diagnosis of ACC includes other salivary gland tumors that exhibit tubular and cribriform structures, such as polymorphic adenocarcinoma, and tumors with basaloid cell morphology, including basal cell adenoma and basal cell adenocarcinoma. The presence of cuboidal or columnar cells containing a pale and oval nucleus and eosinophilic cytoplasm in polymorphous adenocarcinoma contrasts with the hyperchromasia of basaloid cells seen in ACC [10]. Basal cell adenoma can be identified based on the presence of a capsule and the absence of stromal and perineural invasion (PNI) [10], whereas basal cell adenocarcinoma is included in the differential diagnosis of ACC with solid predominance. However, ACC can be distinguished by the presence of palisade peripheral cells showing hyperchromatic nuclei and the absence of prominent mitotic figures and areas of necrosis [10,11]. Other neoplasms of non-glandular origin can also be included in the differential diagnosis of ACC, such as basaloid squamous carcinoma and ameloblastoma, specifically those without obvious stellate cells [10]. The presence of the t (6; 9) (q22–23; p23–24) translocation, which involves the fusion of the MYB oncogene with the NFIB transcription factor, is reported in 80% to 90% of ACC cases and has been used as a diagnostic biomarker [12].

Histopathologically, ACC is characterized by the proliferation of ductal and myoepithelial cells in a tubular, cribriform, or solid pattern [13]. Histopathological evaluation can provide valuable prognostic information, particularly when the solid growth pattern predominates over the tubular and cribriform patterns, which are associated with a less aggressive biological behavior [14,15]. PNI is a relatively common histopathological finding and is associated with a poor prognosis [10]. Four histopathological grading systems have been proposed over time based on the predominance of these features for the determination of prognoses and treatment plans of patients with ACC [14,15,16,17,18]. Other histopathological features such as high-grade transformation (characterized by areas of necrosis, a high mitotic rate, and the loss of biphasic ductal–myoepithelial differentiation), lymphovascular invasion (LVI), presence of tumor necrosis, and positive surgical margins have also been indicated as potential prognostic markers [19,20]. Herein, we review and discuss the impact of histopathological parameters, individual or combined in histopathological grading systems of malignancy, on the prognosis of salivary gland ACC.

## 2. Search and Review Strategies

This is a narrative review. The data included were obtained by searches performed in PubMed and Embase. We attempted to retrieve all studies that explored the association between histopathological features and salivary gland ACC prognosis and that were published in English in peer-reviewed journals from 2015 to July 2023. We used the following keywords, individually or in combination: “adenoid cystic carcinoma” and “salivary gland” and “histopathology” or “histopathological grading” or “solid” or “cribriform” or “tubular” or “histopathological features” and “prognosis” or “management” and “treatment”. The full texts of articles that met our inclusion criteria were read, and the pertinent data are reported in one of the sections of this article.

## 3. Histopathological Pattern and ACC Prognosis

**Figure 1:**
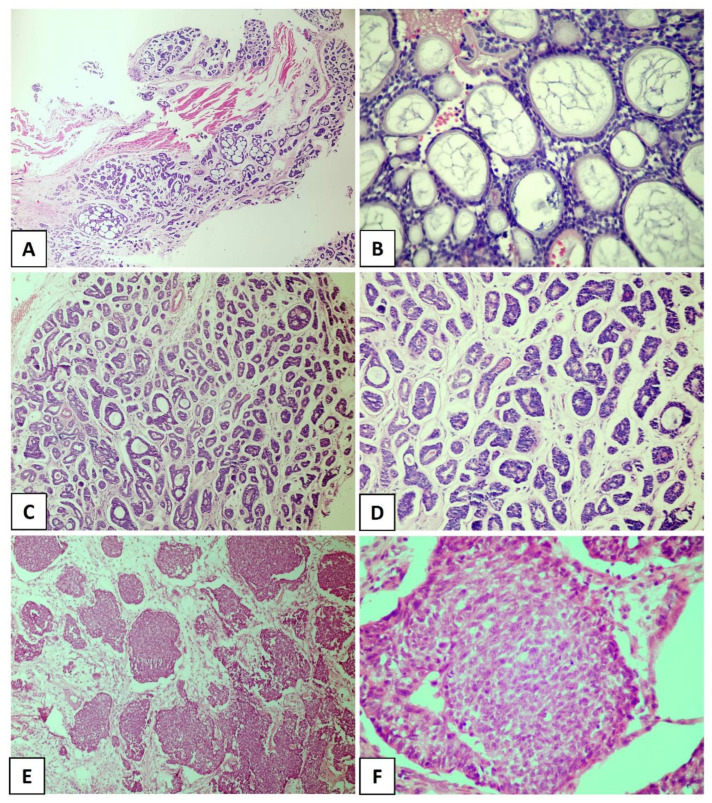
Histological subtypes of the salivary gland adenoid cystic carcinoma. (**A**,**B**) Cribriform pattern, (**C**,**D**) tubular pattern, and (**E**,**F**) solid pattern. ((**A**) In original magnification of 40×, (**C**–**E**) in original magnification of 100×, and (**B**,**F**) in original magnification of 200×).

ACC is characterized by a dual cell population consisting of basaloid cells with myoepithelial/basal differentiation and luminal/epithelial cells, which may histologically acquire specific architectural patterns that determine three pathological subtypes [13,21]. The cribriform pattern is characterized by islands of small or cuboidal basaloid epithelial cells that contain a basophilic nucleus and scarce cytoplasm with multiple pseudocystic spaces filled with basophilic material compatible with glycosaminoglycans (Figure 1A,B). In the tubular pattern, the ductal structures are lined with a single layer of epithelial cells or one or more layers of basaloid cells. An amorphous eosinophilic material may be present in the center of the tubules and the stroma in this pattern is often hyalinized (Figure 1C,D). In the solid pattern, tumor islands are filled with luminal and non-luminal cells dispersed in a dense fibrous connective stroma; areas with ductal or pseudocystic spaces are rare [10,19,22] (Figure 1E,F). Most cases of ACC exhibit more than one pathological pattern [23,24].

For decades, studies have tried to identify histopathological factors that correlate with the prognosis of ACC [14,15,16,17,18]. The solid proliferation of tumor cells (predominance of solid areas) was notoriously one of the first proposed prognostic factors and has been considered a useful histological parameter for assessing survival rates in patients with ACC [14]. Indeed, the solid subtype is considered the most aggressive variant of ACC, which has a poor prognosis [14,25,26,27]. This importance has been confirmed by all histological grading systems for ACC available so far, which are based on the presence and/or proportion of the solid component in the tumor [14,15,16,17,18]. Furthermore, according to the current WHO classification for salivary gland tumors, ACC in which the solid component accounts for more than one-third of the tumor exhibits a poor clinical course [2].

Compared to the cribriform and tubular subtypes, the solid pattern is characterized by high loss of heterozygosity, a larger number of chromosomal and somatic mutations, and high expression of p53 [28,29,30,31,32]. Clinicopathological studies indicate a higher risk of lymph node metastases in solid ACC [14] and a direct association of this pattern with poor overall survival [33]. In addition to these results, de Morais et al. [27] found that the presence of the solid pattern contributed to the negative impact on long-term disease-specific survival (10 years) of cases evaluated in both univariate and multivariate analyses, corroborating the importance of the solid pattern for the prognostic evaluation of ACC.

**Figure 2:**
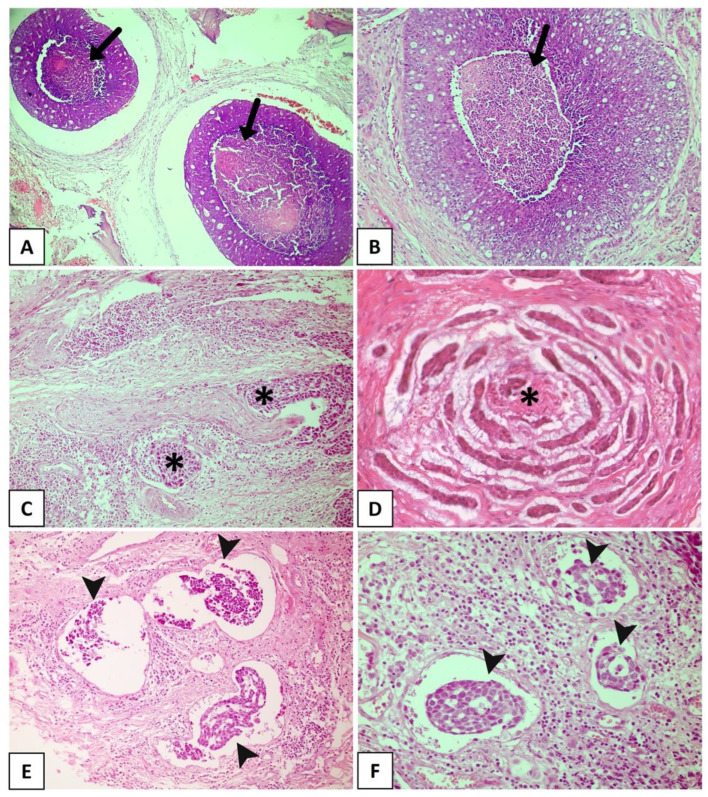
Histopathological features with promising prognostic potential for adenoid cystic carcinoma. (**A**,**B**) The black arrow showing extensive areas of necrosis. Solid subtype with perineural invasion in cords (**C**) and small islands (**D**) (asterisks). (**E**,**F**) Solid tumor with vascular invasion (arrowhead). (Figure (**A**) in original magnification of 40×, (**B**,**C**,**E**,**F**) in original magnification of 100×, and (**D**) in original magnification of 200×).

Some findings that have been described as markers of poor prognosis (Figure 2A,D) are more common in the solid pattern of ACC compared to the cribriform and tubular subtypes, particularly tumor necrosis and high-grade transformation [19,34,35,36]. Xu et al. [19] explored the presence of necrosis as a prognostic factor for ACC in a large retrospective cohort of 135 patients and found that its presence is a predictor of poor recurrence-free survival and disease-free survival. In that study, the presence of necrosis was associated with a high mitotic index and severe nuclear atypia [19]. High-grade transformation, i.e., the presence of a distinct population of anaplastic cells [35], has been histologically defined when the tumor exhibits intense nuclear atypia, a high mitotic index, prominent necrosis, and loss of biphasic ductal-myoepithelial differentiation, features indicative of a poor prognosis [19,34,36]. In a recent study conducted by Zhu et al. [37], high-grade transformation was the only histological parameter associated with shortened overall survival and disease-specific survival in multivariate analysis. It is important to highlight that numerous studies have identified particular mutations in solid tumors. Ferrarotto et al. [38] detected a high frequency of *NOTCH1* mutations among solid ACCs with liver and bone metastasis, as well as a poorer prognosis in these cases. Liu et al. [39] reported that loss of PTEN expression was more frequently seen in ACC compared to other salivary gland malignancies, especially in the poorly differentiated, high-grade subtype of solid ACC.

## 4. Individual Histological Features and ACC Prognosis

Several morphological characteristics were identified as potential prognostic markers for patients with ACC, and the main findings uncovered by this review are reported in Table 1 [14,15,19,40,41,42,43,44,45,46,47].

PNI, which is nowadays considered a consequence of interactions among tumor cells, neural cells, and the perineural niche [27], is found in 48–82% of head and neck ACCs (Figure 2C). It has been widely accepted as the fourth metastatic pathway in ACC, together with the other three well-known routes: direct invasion of surrounding tissues, lymphatic metastasis, and hematogenous metastasis [5,46,47,48]. PNI has been widely reported as an independent negative prognostic factor in head and neck ACC and is significantly associated with decreased overall survival, disease-specific survival, locoregional control, and distant metastasis [20,44,49,50]. Fang et al. [5] reinforced the importance of a careful assessment of PNI in ACC, considering the degree of impairment (invaded layers of the nerve sheath), number of neural invasions, margins of involved nerves (compromised neural circumference), and sites of tumor dissemination (large or small nerves). A meta-analysis conducted by Ju et al. [51] indicated that PNI is strongly associated with poor overall and disease-free survival in ACC. Fordice [52] showed that survival significantly decreases when a large nerve is involved. Invasion involving the perineurium or endoneurium increases the risk of recurrence when compared to tumors whose growth occurs only adjacent to the nerves [53]. In a multicenter cohort of 239 patients with ACC and neural invasion, Amit et al. [54] found intraneural invasion to be an independent negative prognostic marker for overall survival and disease-specific survival. Thus, considering these findings, intraneural invasion is more strongly correlated with a poor prognosis than PNI and is a reliable prognostic predictor of ACC. Considering the association between PNI and ACC prognosis, a comprehensive investigation into the underlying mechanisms related to this process is crucial. This exploration is essential to determine potential predictors of this condition and to identify specific markers that can accurately predict tumor prognosis. Such discoveries could facilitate the development of targeted drugs and effectively reduce the risk of ACC recurrence. However, several studies have highlighted the close correlation between distinct PNI characteristics and factors such as ACC metastasis, locoregional recurrence of the tumor, and overall quality of life of patients [55]. However, as depicted in Table 1, the findings on PNI as a prognostic marker are conflicting.

LVI is another histological finding associated with poor clinical behavior in different malignant neoplasms [42,43,44,56,57,58,59,60]. In ACC (Figure 2E,F), LVI is reported in a large number of cases, ranging from 5% to 70%, and is a known indicator of high recurrence risk and poor prognosis [61,62]. Tang et al. [63] revealed LVI to be an independent prognostic determinant, whereas Oplatek et al. [56] found a significant association with recurrences. In a systematic review and meta-analysis, Martins-Andrade et al. [20] found LVI to be a significant prognostic parameter for head and neck ACC, which was associated with lower overall survival rates and a higher risk of lymph node metastases. The prognostic relevance of LVI as a risk factor for recurrence has been documented in the literature [56,63]. Jeong et al. [60] identified the presence of LVI in surgical pathology specimens to be associated with a reduction in 5-year overall survival in recurrent and metastatic ACC, with multivariate analysis confirming its role as an independent risk factor. Although these results indicate an aggressive evolution associated with LVI, most published studies only described its presence or absence and considered LVI to be a secondary finding. Indeed, few studies investigated the true importance of LVI and its impact on survival in ACC as a primary endpoint. Furthermore, the profile of tumor emboli is not well described, with the types of vessels that are being invaded, whether blood or lymphatic, and the morphological characteristics of the invaded vessel being unknown [9,20]. Therefore, large studies investigating the prognostic potential of LVI in ACC are necessary.

The presence of positive surgical margins has also been proposed as a prognostic factor for ACC [20]. Amit et al. [64] analyzed the role of surgical margins in 507 cases of head and neck ACC in an international multicenter study and found a high rate of positive surgical margins (50% of the analyzed cases). The authors concluded that positive margins are predictors of poor survival in head and neck ACC, while negative margins, corresponding to tumor-free margins <5 mm, are associated with a favorable outcome. Although de Morais et al. [27] found a statistically significant association between positive margins and a poor prognosis at 5 and 10 years in univariate analysis, in multivariate analysis, the status of the surgical margin itself did not have an independent prognostic value. Furthermore, there are no data in the literature that confirm the prognostic role of positive surgical margins in recurrent and metastatic ACC [33,62]. It is worthwhile noting that the ability to achieve large tumor-free margins depends on a number of factors, including tumor location and size, histopathological pattern, and previous treatment [5]. In many cases, surgery is limited by the proximity of vital structures [27], as occurs in some cases of head and neck ACC that have an anatomical propensity for intracranial extension involving vital structures, posing a challenge to achieve negative surgical margins [5]. Studies have shown that patients with ACC arising at sites close to the base of the skull (nasopharynx, nasal cavity, and paranasal sinuses) have a significantly higher risk of local recurrence [64,65].

In a recent study, Xuan et al. [66] proposed an evaluation based on the dominant cell type. The authors applied immunohistochemistry against p63 and CD117 and classified ACCs into epithelial (E-ACC), myoepithelial (M-ACC), and conventional (C-ACC) subtypes. The results showed that the E-ACC subtype was an independent negative prognostic factor for overall survival and recurrence-free survival. According to the authors, the main advantages of this analysis for ACC prognostic prediction are the reduction in subjectivity associated with traditional histological grading, as well as the practicality and simplicity of the method [66].

With respect to prognostic studies, we must consider the inconsistencies of the results reported in the literature, which may be due to many factors such as differences in patient treatment protocols among cohorts, the duration of postoperative follow-up, and the differences in the proportion of patients in different stages of the disease [66,67]. However, despite all these drawbacks, the findings highlight the importance of extensive histological evaluation of the primary tumor to identify the elements that imply greater tumor aggressiveness [62], without disregarding the fact that the intrinsic characteristics of the primary tumor may also influence the outcome of recurrent and metastatic disease [68].

## 5. Histopathological Grading Systems for ACC

In oncology, the development of histopathological grading systems that are associated with clinical aggressiveness and allow accurate differentiation into low- and high-risk tumors is essential and ongoing. A good system should accurately predict the outcome, stratify patients into different treatment categories, use simple parameters that can be easily analyzed, and show minimal inter- and intraobserver variability. In practical terms, this ideal system is difficult to achieve in ACC, mainly because of the scarcity of studies that adequately validate the proposed grading systems. The lack of robust studies can be, in part, attributed to the low prevalence of the tumor and the requirement of a very long follow-up because of the high capacity of late regional and distant recurrence, which can occur even decades after the initial treatment [68,69].

To date, four histopathological grading systems have been proposed for ACC (Table 2). In all systems, the solid component has been established as the main parameter to discriminate the biological aggressiveness of the tumor. Applying the Perzin–Szanto grading system to a series of 218 cases of ACC in the head and neck region, Zhang et al. [49] demonstrated that tumors classified as grade 3 were significantly associated with shorter disease-specific survival and higher recurrence rates than grade 1 or grade 2 tumors. Similarly, Brackrock et al. [70] found poor overall survival for grade 3 tumors in a cohort of 71 ACC cases. However, subsequent studies did not observe an association between the Perzin–Szanto grading system and prognosis and low intra- and interobserver agreement was reported [15,37].

In 2015, Van Weert and collaborators proposed a grading system based exclusively on the presence or absence of the solid component, which was found to be reliable, reproducible, and predictive, with low interobserver variability [14]. Ikawa et al. [69] subsequently corroborated the results of the grading system proposed by van Weert et al. [14] by demonstrating that the presence of solid areas, regardless of their percentage in the tumor, was an independent predictor of recurrence and overall survival. It should be noted that the grading system proposed by van Weert et al. [14] is more objective, and its interobserver variability is lower compared to the systems proposed by Perzin–Szanto and Spiro because it does not require measuring the proportion of the solid component. Furthermore, the van Weert system can be applied in biopsies, at least in those cases in which areas of the solid component are present in tissue, and can be used to guide the most appropriate treatment plan. However, further studies must confirm this potential.

Although all three histopathological grading systems initially proposed for ACC highlighted the importance of the solid tumor component, none of them defined a criterion for the identification of solid areas. Thus, Morita et al. [15] recently proposed the use of the minAmax system, which considers a cut-off of 0.20 mm as the reference (low grade: ≤0.20 mm and high grade: >0.20 mm). In this system, the widest (not lengthiest) solid tumor nest, which should not contain ductal lumen or cystic spaces nor necrotic or hyalinized/eosinophilic areas, is measured. Applying this grading system to 195 salivary gland ACCs, the authors demonstrated better discrimination of the outcomes (overall survival, disease-free survival, and distant metastasis-free survival) with higher hazard ratios and lower 95% confidence intervals compared to other ACC grading systems. Moreover, interobserver reproducibility produced better kappa scores than the other systems [15]. However, this new system has not yet been validated.

## 6. Future Directions

ACC is a challenging clinical entity in terms of treatment because of its unique clinical and pathological features and the lack of prospective data for establishing an optimal treatment approach. Although surgical resection is the preferred primary treatment modality, adjuvant radiotherapy is frequently advocated to improve local control. Data that would guide the appropriate therapeutic protocol for ACC (radiotherapy dosage regimen, prophylactic lymph node dissection, and design of the surgical procedure to obtain tumor-free surgical margins) are still lacking. Chemotherapy is little used for the treatment of ACC, and the results reported so far have not been promising [71]. Many clinical trials have tested chemotherapeutic agents for unresectable ACC diagnosed in advanced stages, recurrent lesions, or when other treatment modalities have not been successful. The results are inconsistent, and further research using new approaches is needed to determine the effectiveness of chemotherapy [71,72]. Thus, the development of methods that help predict the biological behavior of the neoplastic process and guide adequate treatment planning should be encouraged.

There has been increasing interest in recent years in the analysis of biomolecular markers for different neoplastic processes, including ACC. Since it is a simple method with low operational costs, the definition of morphological parameters and grading systems capable of predicting the prognosis and guiding the appropriate treatment should be further explored. Few studies have analyzed the proposed histopathological grading systems, and there is considerable underestimation of their prognostic value despite the promising results reported by different studies. Furthermore, all histopathological grading systems focus solely on the architectural pattern of the tumor. Multicenter studies with long-term follow-up periods are necessary to develop and analyze new histopathological grading systems that include, in addition to the architectural pattern, parameters already known to be of prognostic value such as PNI and LVI. Future research should prioritize exploring less invasive diagnostic methods or diagnostic biopsies that can identify tumors with aggressive behavior. To ensure reproducibility without compromising the prognostic value of variables, it is crucial to standardize the analysis methods for each morphological parameter and to select models that exhibit high inter- and intra-rater agreement.

## 7. Conclusions

ACC is a rare malignancy with aggressive behavior that is accompanied by many surgical and systemic therapeutic challenges. Our review highlights the prognostic role of histopathological findings, including tumor necrosis, high-grade transformation, the epithelial component as dominant cell type, PNI, LVI, and positive surgical margins. Although these histopathological features are consistently associated with ACC outcomes, the existing histopathological grading systems are based solely on the presence or proportion of the solid component or even on the size of the solid tumor nest. Considering that the application of the grading systems associates well with the prognosis of ACC, we suggest that the inclusion of potential histopathological prognostic parameters may not only improve prognostic prediction but also be of clinical significance. In view of the rarity of ACC, large cohort and multicenter studies with a follow-up period of at least 10 years can help to clarify and validate the results already reported in the literature, as well as to identify new prognostic markers.

## Figures and Tables

**Table 1:** Summary of the main features of the articles exploring histopathological features or grading systems in the prognosis of salivary gland adenoid cystic carcinoma.

Authors	Year	Sample Size	Follow-Up in Months, Mean (Range)	Histopathological Parameter or Grading System	Survival	
					Univariable Significance	Multivariable Significance
Bjørndal et al. [40]	2015	201	91 (4–240)	Positive/close margin	Yes	Yes
				Perzin–Szanto system	Yes	Yes
van Weert et al. [14]	2015	87	NI	Perzin–Szanto system	Yes	Yes
				Spiro system	Yes	Yes
				Van Weert system	Yes	Yes
Han et al. [41]	2017	54	68 (16–120)	PNI	Yes	No
				Perzin–Szanto system	Yes	No
Ouyang et al. [42]	2017	228	74.6 (12–288)	LVI	Yes	Yes
				PNI	Yes	No
				Positive/close margin	Yes	Yes
Ouyang et al. [43]	2017	120	60.5 (2–288)	LVI	Yes	Yes
				PNI	Yes	No
				Perzin–Szanto system	Yes	No
Xu et al. [19]	2017	135	75 (1–353)	Perzin–Szanto system	Yes	Yes
Lim et al. [44]	2018	52	72 (5–152)	LVI	Yes	Yes
				PNI	No	No
				Perzin–Szanto system	Yes	Yes
Zhang et al. [45]	2019	158	83.7 (3.5–140)	PNI	Yes	Yes
Akbaba et al. [46]	2020	207	50 (3–121)	PNI	No	No
				Perzin–Szanto system	Yes	Yes
Kawakita et al. [47]	2020	192	65 (5–245)	Positive/close margin	Yes	Yes
				Van Weert system	Yes	Yes
Morita et al. [15]	2021	195	52 (1–263)	Positive/close margin	Yes	Yes
				PNI	No	No
				Perzin–Szanto system	Yes	Yes
				Spiro system	Yes	Yes
				Van Weert system	Yes	Yes
				MinAmax system	Yes	Yes

Legends: PNI, perineural invasion; LVI, lymphovascular invasion; NI, not informed.

**Table 2:** Current histopathological grading systems for salivary gland adenoid cystic carcinoma.

Grading System	Description		
Spiro system [16]	Grade I: typical cribriform pattern exclusively.	Grade II: mixed with substantial solid component.	Grade III: tumors formed mainly by solid areas or with areas of clear anaplasia.
Perzin–Szanto system [17,18]	Grade I: tumors with tubular and cribriform areas, but without solid component.	Grade II: cribriform tumors that were either pure or mixed with <30% of solid areas.	Grade III: tumors with solid component >30%.
van Weert system [14]	Low grade: tumors with absence of solid component (S−).	High grade: tumors with solid areas (S+).	
MinAmax system [15]	Low grade: tumors with complete lack of solid areas or solid areas with diameter ≤0.20 mm.	High grade: tumors with solid nests with diameter >0.20 mm.	

## Data Availability

Not applicable.
